# The Impact of the COVID-19 Pandemic on the Dental Emergency Service from Oradea, Romania: A Retrospective Study

**DOI:** 10.3390/healthcare10091786

**Published:** 2022-09-16

**Authors:** Abel Emanuel Moca, Ioan Andrei Țig, Gabriela Ciavoi, Raluca Iurcov, Lucian Roman Șipoș, Liana Todor

**Affiliations:** Department of Dentistry, Faculty of Medicine and Pharmacy, University of Oradea, 10 Piața 1 Decembrie Street, 410073 Oradea, Romania

**Keywords:** COVID-19, dental emergency, Romania

## Abstract

The COVID-19 pandemic affected the daily lives of the global population, not only in terms of social interaction but also in terms of access to medical and dental care. Non-urgent dental treatments could not be continued during the lockdown and only a small number of dental centres addressed patients with dental emergencies. The aim of this study was to evaluate the socio-demographic characteristics (age, gender, and living environment) of the individuals that accessed the dental emergency centre in Oradea (North-West Romania) and the main causes for accessing the dental emergency service among the population of Oradea (North-West Romania), during the COVID-19 lockdown, between March and May 2020 and, furthermore, to compare the results obtained in the lockdown timeframe (March–May 2020), with the results obtained in the corresponding timeframe in the pre-lockdown year (March–May 2019) and post-lockdown year (March–May 2021). The retrospective study was carried out by analysing the medical records of the patients who were treated in the dental emergency service of the Oradea County Emergency Clinical Hospital in the following periods: March–May 2019, March–May 2020, and March–May 2021. Most patients were treated in 2020, during the lockdown (*n* = 784), predominantly in April (*n* = 308). Most patients treated in April 2020 were male patients (43.7%, *n* = 205) and were aged between 30 and 39 years (19.4%, *n* = 74). The most frequent types of dental emergencies were acute apical periodontitis and acute pulpitis in all the months and years investigated. During the lockdown months of 2020, acute pulpitis was the most frequent type of emergency in March (42.2%, *n* = 100) and May (45.6%, *n* = 109), while in April, acute apical periodontitis was the most frequent type of emergency (43.5%, *n* = 166). The COVID-19 lockdown led to an increase in the number of patients that required emergency treatments and impacted all groups of people investigated.

## 1. Introduction

A patient who requires emergency treatment is an individual who presents with an urgent pathology caused by an illness, an injury, an accident, or a natural disaster, and who needs immediate specialized treatment [1]. In dentistry, situations that require emergency treatment can be of carious, periodontal, or traumatic origin [2]. Dental caries occurs through the interaction between dental structure, oral bacterial plaque that is deposited on the tooth surface, and carbohydrates, in addition to salivary and genetic factors [3]. With a prevalence of up to 52.4% [4], dental caries remains a public health issue [5], which, if left untreated or improperly treated, can lead to complications, such as pulpitis [6], chronic apical periodontitis [7], or dental abscess, which can cause serious general complications, such as meningitis, encephalitis, or even death [8]. Dental trauma mainly affects the upper frontal teeth and permanent teeth, rather than temporary teeth [9], having a complex aetiology that includes interpersonal violence, falls, and traffic accidents [10]. Periodontal emergencies include gingival abscesses, periodontal abscesses, pericoronitis, peri-endo abscesses, necrotizing periodontal diseases, acute herpetic gingivostomatitis, and acute physical, chemical, or thermal injuries [11]. For all dental emergencies, fast and timely intervention is essential to prevent further complications [11].

As an initial response to the pandemic caused by the onset of the coronavirus disease 2019 (COVID-19), lockdowns and social distancing were instituted globally in order to limit the spread of the SARS-CoV-2 virus (severe acute respiratory syndrome coronavirus 2) [12]. The daily life of the global population was affected by the pandemic [13] and dental treatments that were classified as non-urgent could not be continued, given the fact that the activity of dental offices was suspended [14]. At the same time, it was necessary to maintain a dental activity that addressed dental emergencies [12]. Europe became one of the most-affected continents, with high death rates in Italy, Spain, France, and the United Kingdom. In addition to the standard measures, some countries (e.g., Germany) reinforced borders as well [15]. Immediately after instituting the lockdown in the city of Oradea (North-West Romania), the only centre that addressed patients with dental emergencies was the Oradea County Emergency Clinical Hospital, which operated within the Dental Clinic of the Faculty of Medicine and Pharmacy of the University of Oradea. The Romanian College of Dentists considered the following pathologies as emergencies that required urgent treatment: post-extractional haemorrhages, acute pulpitis, acute apical periodontitis, pericoronitis, post-extractional alveolitis, abscesses, cellulitis, mandibular fractures, dislocations of the temporomandibular joint, and ulceronecrotic gingivostomatitis [16].

Considering the fact that the prevalence of caries in Romania is high [17], in the Romanian dental environment, the emphasis falls on the treatment of dental diseases and not on prevention [18] and also that dental visits among the Romanian population are rare in comparison with other European populations [19]; the predisposition to dental emergencies may be higher in the Romanian population. The limited number of dental offices that were authorized to deliver emergency dental services during the COVID-19 lockdown could lead to an overload for the medical staff [20], as well as delays in the presentation to the dental emergency centres, to the point where dental emergencies could cause other complications [21].

The aim of this study was to evaluate the socio-demographic characteristics (age, gender, and living environment) of the individuals that addressed the dental emergency centre in Oradea (North-West Romania), as well as the main reasons for accessing the dental emergency service among the population from Oradea (North-West Romania), during the COVID-19 lockdown between March and May 2020 and to compare the results obtained in the lockdown timeframe (March–May 2020) with the results obtained in the corresponding timeframe in the pre-lockdown year (March–May 2019) and post-lockdown year (March–May 2021).

## 2. Materials and Methods

### 2.1. Ethical Considerations

The study received approval from the Ethical Committee of the Oradea County Emergency Clinical Hospital (IRB No. 22143/06.07.2022) and it was carried out according to the principles imposed by the Helsinki Declaration of 2008 and its later amendments. Upon presentation to the dental emergency service, adult patients (over 18 years old) signed an informed consent whereby they agreed that their medical data could be used anonymously for future scientific research. For underage patients (under 18 years of age), the informed consent for the anonymous use of medical data was signed by parents or legal guardians.

### 2.2. Participants and Data Collection

The retrospective study was carried out by analysing the medical records of the patients who were treated in the dental emergency service of the Oradea County Emergency Clinical Hospital. This is a free public service that operates in the building of the Faculty of Medicine and Pharmacy of the University of Oradea. At the beginning of the lockdown period, this was the only centre that treated dental emergencies in Oradea. In this study, the medical records of the patients who were treated in the dental emergency service in the following periods were analysed: 1 March–31 May 2019 (pre-lockdown timeframe), 1 March–31 May 2020 (lockdown timeframe), and 1 March–31 May 2021 (post-lockdown timeframe).

Initially, all patients who presented themselves to the dental emergency service of the Oradea County Emergency Clinical Hospital were included in the study. Patients for whom necessary information was missing from the medical records (gender, age, living environment, type of emergency) were excluded.

The following variables were taken into account: gender (male, female), age (0–9 years, 10–19 years, 20–29 years, 30–39 years, 40–49 years, 50–59 years, 60–69 years, 70–79 years, 80–89 years, 90–99 years), living environment (urban, rural), and the type of emergency for which they used the dental emergency service (acute apical periodontitis, abscess, acute pulpitis, trauma, and periodontal emergencies).

In order to avoid bias, the patients’ medical records were double checked by the author who collected the data, but also by the author who was responsible for compiling the statistics.

### 2.3. Statistical Analysis

The statistical analysis was performed using IBM SPSS Statistics 25 (IBM, Chicago, IL, USA) and Microsoft Office Excel/Word 2013 (Microsoft, Redmond, WA, USA). Qualitative variables were expressed in absolute form or as percentages and were compared using Fisher’s Exact or Pearson Chi-Square tests. Z-tests with Bonferroni correction were performed to detail the results obtained in the contingency tables.

## 3. Results

**Figure 1:**
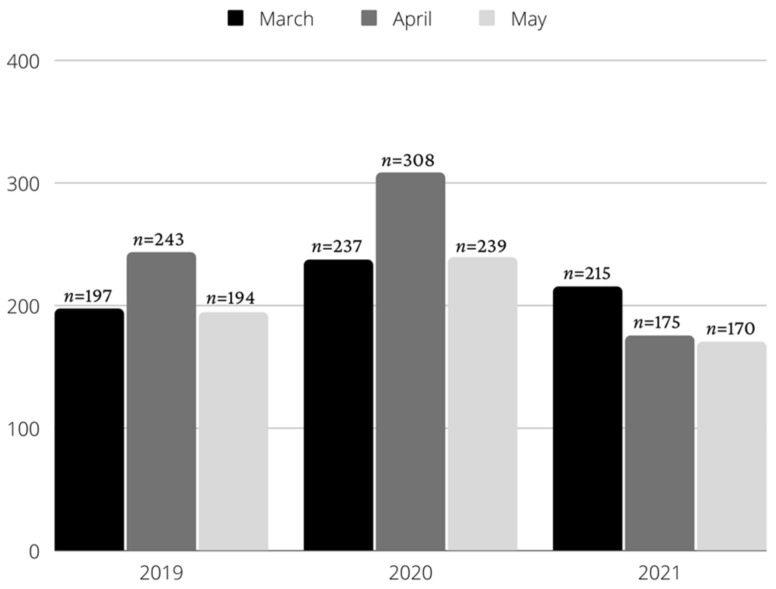
Evolution in 2019, 2020, and 2021.

In total, 2113 medical files were registered in the investigated periods and 135 were lacking information regarding age (*n* = 22), gender (*n* = 31), living environment (*n* = 34), type of emergency (*n* = 13), or had no informed consent (*n* = 35). A final number of 1978 patients were included in the study, including 634 for 2019, 784 for 2020, and 560 for 2021. The distribution of patients according to the different investigated months is shown in Figure 1.

### 3.1. Gender, Living Environment, Age, and Type of Emergency

**Figure 2:**
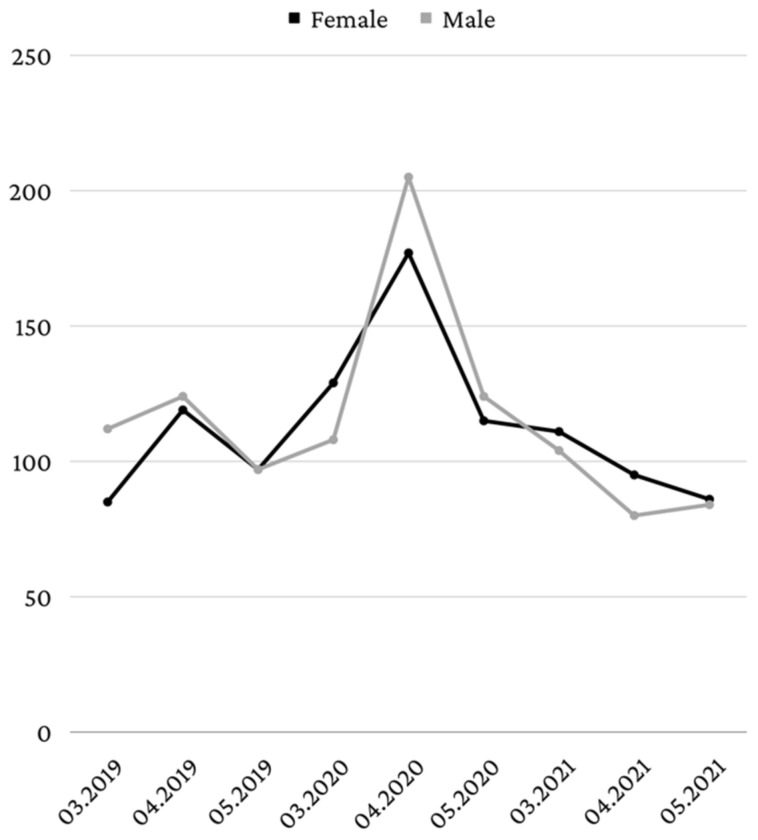
Distribution according to gender.

The data in Figure 2 represent the distribution of patients who came to the dental emergency service in the months of March, April, and May 2019, 2020, and 2021, according to their gender. In March 2019, the majority of patients was male (56.9%, *n* = 112), a situation similar to that of April 2019, when 51% (*n* = 124) of the patients were male. In May 2019, the gender distribution was equal. In March 2020, 54.4% (*n* = 129) of the patients were female and in April and May 2020, male patients predominated (53.7%, *n* = 205—April; 51.9%, *n* = 124—May). In the three investigated months of 2021, female patients predominated (51.6%, *n* = 111—March; 54.3%, *n* = 95—April; 50.6%, *n* = 86—May).

**Figure 3:**
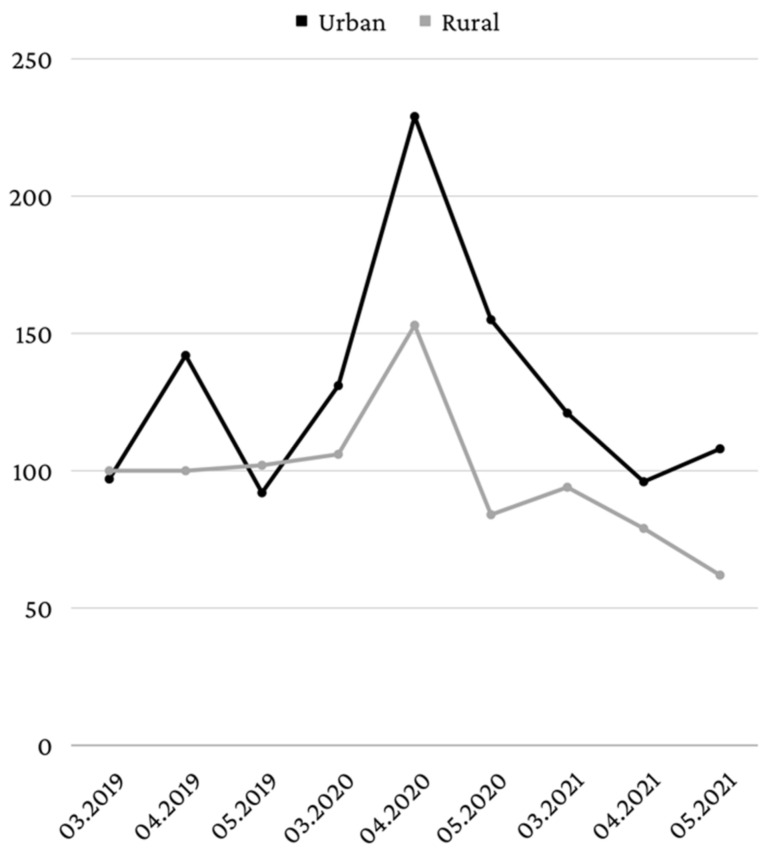
Distribution according to the living environment.

In terms of patient distribution by living environment, in March 2019 and May 2019, a slight predominance of patients from rural areas was identified (50.8%, *n* = 100—March; 52.6%, *n* = 102—May), but in April 2019, patients from the urban environment predominated (58.4%, *n* = 142). In the period from March to May 2020, patients from the urban environment predominated, in all 3 investigated months. Thus, in March 2020, 55.3% (*n* = 131) of the patients came from the urban environment, in April 2020, 59.9% (*n* = 229), and in May 2020, 64.9% (*n* = 155). In 2021, patients from the urban environment predominated in all 3 months, as follows: 56.3% (*n* = 121) in March, 54.9% (*n* = 96) in April, and 63.5% (*n* = 108) in May, a situation similar to that in 2020 (Figure 3).

The data in Table 1 show the distribution of patients according to the different age groups. In 2019, in March, patients aged between 20 and 29 years predominated (24.9%, *n* = 49) and in the months of April and May, the largest number of patients was aged between 0 and 9 years (24.7%, *n* = 60—April; 25.8%, *n* = 50—May). In 2020, patients from the 20–29 age group predominated in March (19%, *n* = 45) and May (18%, *n* = 43), while in April, patients from the 30–39 age group predominated (19.4%, *n* = 74). In 2021, the largest number of patients who came for emergency treatment was from the 20–29 age group, in March (22.8%, *n* = 49), April (28%, *n* = 49), and May (23.5%, *n* = 40).

The data in Table 2 show the distribution of patients according to the type of emergency with which they presented to the dental emergency service. In all 3 years, the most frequent emergencies were acute apical periodontitis and acute pulpitis. In the months of March 2019, April 2020, March 2021, and May 2021, acute apical periodontitis predominated, while in all other investigated months, acute pulpitis predominated.

### 3.2. Comparisons between 2019, 2020, and 2021

The data in Table 3 represent the evolution in the month of March in the investigated years, related to the variables investigated in the study. According to the Pearson Chi-Square tests, the differences between years in relation to gender (*p* = 0.055) and living environment (*p* = 0.303) were not significant. However, the results showed that:

- The differences between the frequency of age groups and years were significant (*p* = 0.011) and Z tests with Bonferroni correction showed that patients aged 0–9 years were more frequent in March 2019 (21.3%) than in 2021 (9.8%) and patients aged 40–49 were more frequent in March 2020 (16.9%) than in 2019 (8.1%);
- The differences between the frequency of the different types of emergencies and the years were significant (*p* = 0.009) and Z tests with Bonferroni correction showed that abscesses were more frequent in March of 2019 (12.7%) than in March 2021 (5.1%).

Regarding the evolution in the month of April in the investigated years, related to the variables investigated in the study, according to the Pearson Chi-Square tests, the differences between years related to gender (*p* = 0.219), living environment (*p* = 0.527), and type of emergency (*p* = 0.060) were not significant. However, the results showed that the differences between the frequency of age groups and years were significant (*p* < 0.001), and Z tests with Bonferroni correction showed the following:

- Patients aged 0–9 years were more frequent in April 2019 (24.7%) than in April 2020 (11.3%) or 2021 (10.9%);
- Patients aged 20–29 years were more frequent in April 2021 (28%) than in April 2020 (18.6%);
- Patients aged between 60 and 69 years/70 and 79 years were more frequent in April 2020 (8.9%/3.9%) than in April 2019 (3.3%/0%) (Table 4).

The data in Table 5 represent the evolution in the month of May in the investigated years related to the variables investigated in the study. According to the Pearson Chi-Square tests, the differences between years related to gender (*p* = 0.869) and type of emergency (*p* = 0.088) were not significant. The results showed that the differences between the frequency of age categories and years were significant (*p* < 0.001) and Z tests with Bonferroni correction showed the following:

- Patients aged between 0 and 9 years old were more frequent in May of 2019 (25.8%) than in May 2020 (14.6%);
- Patients aged 40–49 were more frequent in May of 2020 (14.2%) or May 2021 (15.9%) than in May 2019 (5.2%);
- Patients aged 50–59 were more frequent in May 2020 (10%) than in May 2019 (3.6%).

The differences between the frequency of the living environments and years were significant (*p* = 0.009) and Z tests with Bonferroni correction showed that patients from rural areas were more frequent in May of 2019 (52.6%) than in May 2020 (35.1%) or May 2021 (36.5%), while patients from urban areas were more frequent in May of 2020 (64.9%) or May 2021 (63.5%) than in May 2019 (47.4%).

## 4. Discussion

The COVID-19 pandemic had an impact on the emergency dental service within the Oradea County Emergency Clinical Hospital, the number of patients who received emergency treatment being higher in 2020 compared to 2019 or 2021. This was caused, most likely, by the fact that during the lockdown, elective dental care was suspended [22]. The lockdown was initiated in different countries starting from March 2020 [23] and, in Romania, the lockdown period lasted from 21 March 2020 to 15 May 2020 [24]. During this period, patients with dental emergencies were treated only in centres that were approved for delivering emergency treatments. The increased number of patients who presented themselves for emergency treatment between March 2020 and May 2020 is similar to studies from other populations. Thus, during the lockdown period, an increase in the number of patients who came to the emergency dental services for the treatment of severe abscesses was observed [25]. An overall increase in the total number of patients who presented themselves for emergency treatment was reported in the city of Cluj-Napoca, Romania, as well [26].

Despite the fact that, in Romania, the lockdown period was between 21 March 2020 and 15 May [24], the recommendations for social distancing and preventive measures were introduced earlier and lasted beyond the lockdown period. For this reason, including all patients who were treated between 1 March and 31 March in the years 2019, 2020, and 2021 in this study was considered useful. In this way, it was possible to analyse the situation for 3 whole months. We chose to analyse only these 3 months (March, April, and May) because they were affected by the lockdown period [27]. Of all the months analysed, April 2020 was the most demanding, with 308 treated patients, but a similar number of patients were treated in April 2019 (*n* = 243). However, in April 2021, only 175 patients were treated.

For the completion of this study, the dental emergency pathologies with which the patients presented with were divided into five categories of dental emergencies, as follows: acute apical periodontitis, acute pulpitis, abscess, trauma, and periodontal emergencies. The most-frequent types of dental emergencies during the lockdown were acute apical periodontitis (in April 2020) and acute pulpitis (in March and May 2020). This is due to the fact that, usually, the complications of dental caries are responsible for most dental emergencies [28]. In the trauma category, we included avulsion, intrusion, extrusion, dislocation, subluxation, crown fracture, and root fracture [29]. Facial fractures that affected the midface or mandible were not included because their treatment was not carried out in the emergency dental service where this study took place but in the Maxillofacial Surgery Department of the Oradea County Emergency Clinical Hospital. The incidence of traumatic injuries was low in all the investigated months, ranging from 2.1% (May 2019) to 6.5% (March 2021). During the 2020 lockdown, the incidence was similar in all the 3 months, hovering around the percentage of 5.5%. This incidence was similar to other studies that analysed the presentation in the dental emergency service in pre-lockdown, lockdown, and post-lockdown [30]. In the category of periodontal emergencies, we included post-extraction haemorrhages, pericoronitis, post-extraction alveolitis, and ulceronecrotic gingivostomatitis, because the recommendations of the Romanian College of Dentists [16] were taken into account. They had a relatively low incidence during the lockdown period, which varied from 3.4% to 6.3%, but were higher in the post-lockdown period, with an incidence that varied from 5.9% to 10.7%. This aspect could be explained by the fact that in the post-lockdown period, dental offices were reopened and routine dental treatments, such as extractions, could be performed again. Among the most-frequent complications that can occur after dental extractions are post-extractional pain and haemorrhage [31], which were included in the category of periodontal emergencies in the present study.

Male patients were more numerous in April 2020 and May 2020. This can be explained by the fact that, usually, male patients go to the dentist less often, do not use dental floss as frequently as female patients, and have poorer oral healthcare habits [32]. Likewise, male patients are more reluctant to seek medical services, even for the treatment of general illnesses [33], probably due to a sense of stoicism and self-reliance [34,35]. However, between March and May 2021, more female patients were treated in the emergency department, but their number was lower compared to that of male patients treated in the period March–May 2020. In most months, patients from the urban environment were predominant, pre-lockdown, during lockdown, and post-lockdown. Between March 2020 and May 2020, the percentage of the patients living in the urban environment increased from 55.3% in March to 64.9% in May. The higher number of patients from the urban environment may be caused by the fact that the rural population tends to have poor use of healthcare services, including the use of dental healthcare services [36]. Another reason could be the fact that in Romania, most people live in the urban environment, with 53.8% of the country’s population living in cities [37].

The patients treated between March 2020 and May 2020 were mainly from the 20–29 years age group (March and May) and from the 30–39 years group (April). However, children or adolescent patients represented an important percentage of the total number of treated patients. Thus, in March 2020, 77 children and adolescents (32.5%) were treated, in April 2020, 90 children and adolescents (23.6%) were treated, and in May 2020, 73 children and adolescents (30.5%) were treated. However, between March 2019 and May 2019, the percentage of children and adolescents treated was even higher, ranging from 42.4% (April 2019) to 50.5% (May 2019). In Romania, the prevalence of caries in children and adolescents is high. A study conducted by Funieru et al. (2014) reported a 64% incidence of untreated dental caries among children and adolescents aged between 10 and 17 years [38]. A high prevalence of caries in children aged 6 years old from Romania, with affected deciduous teeth, was also reported by Dumitrescu et al. (2022) [39]. This high prevalence is influenced not only by the socioeconomic background and parental education, but also by dietary preference for sugary foods [40]. The fewest patients treated in the dental emergency service were from the following age groups: 60–69 years, 70–79 years, 80–89 years, and 90–99 years. Thus, in March 2020, 12 elderly patients were treated (5%), in April 2020, 49 patients were treated (12.8%), and in May 2020, 25 elderly patients were treated (10.4%). The percentages were low in March–May 2019 and March–May 2021 as well. In general, the number of edentulous patients in Romania increases with age, so that beginning with the age of 60 years, the incidence of edentulism is high [41].

The impact of the COVID-19 pandemic and of the lockdowns on dental emergency services was also investigated by Petrescu et al. (2020). The authors compared the presentation in the dental emergency service in Cluj-Napoca in April 2019 and April 2020, pre-lockdown and during lockdown, and identified an increase in cases treated in April 2020 [26]. The increase in cases treated during the lockdown period was also identified in this study.

The present study analysed the periods March–May 2019 (pre-lockdown), March–May 2020 (during lockdown), and March–May 2021 (post-lockdown), both separately and comparatively. Up until the moment of the design of this study, no similar studies (investigating 3 years) conducted in the Romanian population were identified.

In order to limit the spread of the virus in dental offices and dental clinics, treatment and nontreatment areas should be separated and copper should be used for surfaces that are most-frequently touched. At the same time, all waste management regulations should be strictly respected [42].

The study has, however, some limitations. Firstly, this is a retrospective study, so the data available in the medical files may be incorrectly or incompletely provided at the time of examination. Secondly, the emergency diagnostics were based on examination and information obtained from patients and the symptomatology could be misreported.

## 5. Conclusions

The number of patients treated in March–May 2020 (during lockdown) was higher than the number of patients treated in March–May of 2019 (pre-lockdown) and 2020 (post-lockdown). Despite the increase in the number of patients treated in the dental emergency centre during the COVID-19 pandemic, a large number of patients was observed in 2019 and 2021 as well. This suggests the need to develop better oral care education programs for patients. The need for thorough oral prevention must be emphasized to all patients.

## Figures and Tables

**Table 1:** Distribution according to age group.

Date/Age Group	0–9 (*n*, %)	10–19 (*n*, %)	20–29 (*n*, %)	30–39 (*n*, %)	40–49 (*n*, %)	50–59 (*n*, %)	60–69 (*n*, %)	70–79 (*n*, %)	80–89 (*n*, %)	90–99 (*n*, %)
**03.2019**	42 (21.3%)	44 (22.3%)	49 (24.9%)	33 (16.8%)	16 (8.1%)	10 (5.1%)	2 (1%)	1 (0.5%)	-	-
**04.2019**	60 (24.7%)	43 (17.7%)	52 (21.4%)	37 (15.2%)	30 (12.3%)	12 (4.9%)	8 (3.3%)	1 (0.4%)	-	-
**05.2019**	50 (25.8%)	48 (24.7%)	46 (23.7%)	25 (12.9%)	10 (5.2%)	7 (3.6%)	7 (3.6%)	1 (0.5%)	-	-
**03.2020**	37 (15.6%)	40 (16.9%)	45 (19%)	42 (17.7%)	40 (16.9%)	21 (8.9%)	8 (3.4%)	2 (0.8%)	2 (0.8%)	-
**04.2020**	43 (11.3%)	47 (12.3%)	71 (18.6%)	74 (19.4%)	60 (15.7%)	38 (9.9%)	34 (8.9%)	15 (3.9%)	-	-
**05.2020**	35 (14.6%)	38 (15.9%)	43 (18%)	40 (16.7%)	34 (14.2%)	24 (10%)	15 (6.3%)	8 (3.3%)	2 (0.8%)	-
**03.2021**	21 (9.8%)	42 (19.5%)	49 (22.8%)	41 (19.1%)	22 (10.2%)	24 (11.2%)	11 (5.1%)	2 (0.9%)	2 (0.9%)	1 (0.5%)
**04.2021**	19 (10.9%)	23 (13.1%)	49 (28%)	22 (12.6%)	26 (14.9%)	14 (8%)	15 (8.6%)	6 (3.4%)	1 (0.6%)	-
**05.2021**	28 (16.5%)	28 (16.5%)	40 (23.5%)	23 (13.5%)	27 (15.9%)	12 (7.1%)	8 (4.7%)	2 (1.2%)	2 (1.2%)	-

*n*—number; %—percentage.

**Table 2:** Distribution according to type of emergency.

Date/Emergency	Acute Apical Periodontitis (*n*, %)	Abscess (*n*, %)	Acute Pulpitis (*n*, %)	Trauma (*n*, %)	Periodontal Emergency (*n*, %)
**03.2019**	90 (45.7%)	25 (12.7%)	68 (34.5%)	5 (2.5%)	9 (4.6%)
**04.2019**	85 (35%)	31 (12.8%)	97 (39.9%)	12 (4.9%)	18 (7.4%)
**05.2019**	74 (38.1%)	22 (11.3%)	88 (45.4%)	4 (2.1%)	6 (3.1%)
**03.2020**	85 (35.9%)	24 (10.1%)	100 (42.2%)	13 (5.5%)	15 (6.3%)
**04.2020**	166 (43.5%)	30 (7.9%)	152 (39.8%)	21 (5.5%)	13 (3.4%)
**05.2020**	83 (34.7%)	19 (7.9%)	109 (45.6%)	13 (5.4%)	15 (6.3%)
**03.2021**	85 (39.5%)	11 (5.1%)	82 (38.1%)	14 (6.5%)	23 (10.7%)
**04.2021**	67 (38.3%)	12 (6.9%)	79 (45.1%)	6 (3.4%)	11 (6.3%)
**05.2021**	75 (44.1%)	20 (11.8%)	57 (33.5%)	8 (4.7%)	10 (5.9%)

*n*—number; %—percentage.

**Table 3:** Evolution for March 2019, 2020, and 2021 according to the investigated variables.

March				
	2019	2020	2021	*p* *
Gender				
Female	85	129	111	0.055
Male	112	108	104	
**Age group**				
0–9 y	42	37	21	0.011
10–19 y	44	40	42	
20–29 y	49	45	49	
30–39 y	33	42	41	
40–49 y	16	40	22	
50–59 y	10	21	24	
60–69 y	2	8	11	
70–79 y	1	2	2	
80–89 y	0	2	2	
90–99 y	0	0	1	
**Living environment**				
Rural	100	106	94	0.303
Urban	97	131	121	
**Type of emergency**				
Acute apical periodontitis	90	85	85	0.009
Abscess	25	24	11	
Acute pulpitis	68	100	82	
Trauma	5	13	14	
Periodontal emergency	9	15	23	

* Pearson Chi-Square Test.

**Table 4:** Evolution for April 2019, 2020, and 2021 according to the investigated variables.

April				
	2019	2020	2021	*p* *
Gender				
Female	119	177	95	0.219
Male	124	205	80	
**Age group**				
0–9 y	60	43	19	<0.001
10–19 y	43	47	23	
20–29 y	52	71	49	
30–39 y	37	74	22	
40–49 y	30	60	26	
50–59 y	12	38	14	
60–69 y	8	34	15	
70–79 y	1	15	6	
80–89 y	0	0	1	
**Living environment**				
Rural	101	153	79	0.527
Urban	142	229	96	
**Type of emergency**				
Acute apical periodontitis	85	166	67	0.060
Abscess	31	30	12	
Acute pulpitis	97	152	79	
Trauma	12	21	6	
Periodontal emergency	18	13	11	

* Pearson Chi-Square Test.

**Table 5:** Evolution for May 2019, 2020, and 2021 according to the investigated variables.

May				
	2019	2020	**2021**	***p*** *
Gender				
Female	97	115	86	0.869
Male	97	124	84	
**Age group**				
0–9 y	50	35	28	<0.001
10–19 y	48	38	28	
20–29 y	46	43	40	
30–39 y	25	40	23	
40–49 y	10	34	27	
50–59 y	7	24	12	
60–69 y	7	15	8	
70–79 y	1	8	2	
80–89 y	0	2	2	
**Living environment**				
Rural	102	84	62	<0.001
Urban	92	155	108	
**Type of emergency**				
Acute apical periodontitis	74	83	75	0.088
Abscess	22	19	20	
Acute pulpitis	88	109	57	
Trauma	4	13	8	
Periodontal emergency	6	15	10	

* Pearson Chi-Square Test.

## Data Availability

The data presented in this study are available on request from the corresponding authors. The data are not publicly available due to privacy reasons.
